# Anti-cariogenic Properties of *Lactobacillus plantarum* in the Utilization of Galacto-Oligosaccharide

**DOI:** 10.3390/nu15092017

**Published:** 2023-04-22

**Authors:** Xinyan Huang, Jianhang Bao, Yan Zeng, Gina Meng, Xingyi Lu, Tong Tong Wu, Yanfang Ren, Jin Xiao

**Affiliations:** 1 Eastman Institute for Oral Health, University of Rochester Medical Center, Rochester, NY 14642, USA; xinyan_huang@urmc.rochester.edu (X.H.); jianhang_bao@urmc.rochester.edu (J.B.); yan_zeng@urmc.rochester.edu (Y.Z.); 2 School of Stomatology, Henan University, Zhengzhou 450046, China; 3 School of Arts and Science, University of Rochester, Rochester, NY 14627, USA; 4 Department of Biostatistics and Computational Biology, University of Rochester Medical Center, Rochester, NY 14642, USA

**Keywords:** Galacto-oligsaccharide, caries, antifugal, *Lactobacillus plantarum*, *Streptococcus mutans*

## Abstract

Ecological approaches can help to correct oral microbial dysbiosis and drive the advent and persistence of a symbiotic oral microbiome, which benefits long-term dental caries control. The aim of this study was to investigate the impact of the prebiotic Galacto-oligosaccharide (GOS) on the growth of probiotics *L. plantarum* 14,917 and its effect on the inhibitory ability of *L. plantarum* 14,917 against the growth of *Streptococcus mutans* and *Candida albicans* in an in vitro model. Single-species growth screenings were conducted in TSBYE broth with 1% glucose and 1–5% GOS. Interaction experiments were performed using duo- and multi-species models with inoculation of 10^5^ CFU/mL *S. mutans*, 10^3^ CFU/mL *C. albicans*, and 10^8^ CFU/mL *L. plantarum* 14,917 under 1%, 5% GOS or 1% glucose. Viable cells and pH changes were measured. Real-time PCR was utilized to assess expression of *C. albicans* and *S. mutans* virulence genes. Six replicates were used for each group. Student’s *t*-test, one-way ANOVA, and Kruskal-Wallis were employed to compare the outcomes of different groups. GOS significantly inhibited the growth of *C. albicans* and *S. mutans* in terms of growth quantity and speed when the two strains were grown individually. However, GOS did not affect the growth of *L. plantarum* 14,917. Moreover, 1% and 5% GOS enhanced the anti-fungal performance of *L. plantarum* 14,917 in comparison to 1% glucose. GOS as the carbon source resulted in a less acidic environment in the *C. albicans* and *S. mutans* duo-species model and multispecies model where *L. plantarum* 14,917 was added. When GOS was utilized as the carbohydrate substrate, *S. mutans* and *C. albicans* had a significant reduction in the expression of the *HWP1*, *ECE1*, *atpD*, and *eno* genes (*p* < 0.05). To our knowledge, this is the first study that reported the ability of GOS to neutralize *S. mutans-C. albicans* high caries of medium pH and to disrupt virulence gene expression. Moreover, as a prebiotic, GOS augmented the inhibitory ability of *L. plantarum* against *C. albicans* in vitro. The current study revealed the anti-caries potential of prebiotics GOS and shed light on novel caries prevention strategies from the perspective of prebiotics and probiotics. These findings provide a rationale for future biofilm or clinical studies to elucidate the effect of GOS on modulating oral microbiota and caries control.

## 1. Introduction

Dental caries are transmissible and biofilm-dependent infectious diseases. They could cause acute/chronic pain and diminish the quality of life of those involved [1,2]. The imbalance of oral microbial as one of the caries etiologies has been well acknowledged [3]. *Streptococcus mutans* is a well known pathogenic microorganism for dental caries with strong acidogenic and aciduric capacities [4,5]. Moreover, *S. mutans* can adhere to the tooth surface through the production of extracellular glucans via glucosyltransferases [5]. *Candida albicans* is a fungus commonly found in oral cavities of children and root caries. Children with oral *C. albicans* have been found to have higher odds of experiencing early childhood caries (ECC) compared to children without *C. albicans* [6]. The toxicity of plaque-biofilms is amplified by symbiotic cross-kingdom interactions between *S. mutans* and *C. albicans* in multiple in vivo and in vitro studies [7,8,9]. Furthermore, this kind of interaction can potentially enhance biofilm resistance to human saliva [8] with glucosyltransferases B (*GtfB*), a bacterial exoenzyme from *S. mutans*, which unevenly, but stably, binds to mannoproteins on the *C. albicans* surface.

A healthy oral microbiome plays a crucial role in controlling oral diseases, such as dental caries and periodontal disease. Ecological approaches can help correct microbial dysbiosis and drive the advent and persistence of a symbiotic oral microbiome, which benefits long-term caries control [10]. As one of the probiotic *Lactobacilli* species, *Lactobacillus* *plantarum* had an inhibitory effect on the growth of *C. albicans* and *S. mutans* in planktonic and biofilm conditions and in animal models [11,12,13,14]. However, ideal conditions that facilitate and maximize the role of *L. plantarum* remain to be elucidated. Previous studies commonly used sucrose as the sugar resource to simulate and trigger high-risk conditions of dental caries. However, it is not known how the role of *L. plantarum* will be affected by using different sugary substrates.

Galacto-oligosaccharides (GOSs) are chains of galactose molecules with multiple health benefits. GOS is a type of prebiotic that is defined as “a substrate which is selectively utilized by the host gut microflora which confers health benefit” [15]. It can enhance the growth and activity of probiotics selectively [16]. GOS is presently one of the most favored prebiotics used in commercial infant formula to imitate the beneficial effects of the human milk oligosaccharides (HMOs) in breast milk [17]. Infants fed with GOS-enriched formula have a comparable development gut microbiome to those fed by breast milk [18,19]. Several studies have reported the beneficial effects of GOS on the intestinal system [20,21]. The benefits include acting as soluble decoy receptors to prevent the adhesion of pathogens to epithelial cells, stimulating tight junctions, and enhancing intestinal barrier function via modulation of goblet cells [20].

*L. plantarum* is capable of utilizing GOS as a source of energy. Previous research has shown that consuming GOS can help to increase the levels of beneficial bacteria in the gut, including *L. plantarum;* however, the utilization of prebiotics and the effect of prebiotics on the growth of *L. plantarum* differs by strains [22,23,24,25,26]. A recent study assessed the utilization of GOS by 21 *L. plantarum* strains. Additionally, all *L. plantarum* strains grew relatively well in media supplemented with GOS [23].

To our best knowledge, until now, no studies have reported whether GOS could enhance or reduce the inhibitory ability of *L. plantarum* on the growth of *C. albicans* and *S. mutans.* The exact impact of GOS on the inhibitory ability of *L. plantarum* may depend on various factors, such as the specific strain of *L. plantarum*, the concentration of GOS, and the growth conditions. The current study aimed to investigate the impact of prebiotics GOS on the growth of probiotics *L. plantarum* 14,917 and the GOS’s effect on the inhibitory ability of *L. plantarum* 14,917 against the growth of *S. mutans* and *C. albicans* in an in vitro model. This study may stimulate greater interest in the use of synbiotics, prebiotics, and probiotics in dentistry and justify additional research into the efficacy of GOS and *L. plantarum* 14,917 on caries prevention using a multi-species biofilm model in vitro and through clinical investigations.

## 2. Materials and Methods

### 2.1. Microorganisms and GOS

The microorganisms used in this study are lab strains, *S. mutans UA159*, *C. albicans* 5314, and *L. plantarum* ATCC 14,917. We selected *L. plantarum* ATCC 14,917 because the effectiveness of this strain on inhibiting the growth of *S. mutans* and *C. albicans* in planktonic or multispecies biofilms models had been verified previously by us [14,27]. The GOS used in this study was purchased from BOS Science (New York, NY, USA).

### 2.2. Starter Preparation

YPD agar (BD Difco™, San Jose, CA, USA, 242,720), blood agar (TSA with sheep blood, Thermo Scientific™, Waltham, MA, USA, R01202), and MRS agar (BD Difco™, 288,210) were used to recover *C. albicans*, *S. mutans*, and *L. plantarum* from frozen stock, respectively. Following a 48-h incubation period, overnight incubation (5% CO_2_, 37 °C) was conducted by inoculating three to five colonies of each species into 10 mL of broth. YPD broth (BD Difco™, 242,820) was used to grow *C. albicans*; TSBYE broth (3% Tryptic Soy, 0.5% Yeast Extract Broth, BD Bacto™ 286,220 and Gibco™ 212,750) with 1% (*w*/*v*) glucose was used to grow *S. mutans*; and *L. plantarum* was cultivated in MRS broth (BD Difco™, 288,130). On the following day, 0.5 mL of the overnight starters were added to individual glass tubes containing fresh broth and incubated for 3–4 h to reach the mid-exponential phase with desirable optical density (OD). The morning starters were then prepared for the subsequent establishment of growth screening assays and planktonic models delineated below.

### 2.3. Screening for Microorganisms’ Growth in GOS

The culture medium was prepared with TSBYE broth and supplemented with 1–5% (*w*/*v*) GOS or 1% glucose as control groups. The morning starters (with desirable OD) of *S. mutans*, *C. albicans*, and *L. plantarum* were diluted with the culture medium prepared above to reach the desirable concentration, respectively (*C. albicans* of 10^5^ CFU/mL, *S. mutans*, and *L. plantarum of* 10^8^ CFU/mL). An amount of 200 µL of culture of each species with different culture medium were distributed in the 96-well plates (Corning, Inc., Corning, NY, USA) and incubated for 20 h at 37 °C in ten replicates. Non-inoculated wells were included as blank controls in triplicate. The OD at 600 nm (OD600) and pH value were measured every 1 h for the first 8 h and at 20 h. During the exponential phase, maximum specific growth rates (µmax) were calculated through linear regressions of the plots of ln (OD600) versus time [24].

### 2.4. Planktonic Model

The starting concentrations of *S. mutans* (10^5^ CFU/mL) and *C. albicans* (10^3^ CFU/mL) were selected to simulate a clinical setting with high-caries risk. The inoculation quantity of *L. plantarum* (10^8^ CFU/mL) was chosen because it was the working concentration of *L. plantarum* 14,917 that demonstrated inhibitory effects on the growth of *S. mutans* and *C. albicans* based on the previous study [14]. Single-, duo-, and multi-species conditions of *S. mutans*, *C. albicans*, and *L. plantarum* were cultivated in 10 mL TSBYE broth with 1%, 5% GOS, or 1% glucose for 20 h (5% CO_2_, 37 °C). Microorganisms grown in TSBYE broth with 1% glucose were used as the control. The assessment of microbial growth was conducted at 0, 6, and 20 h through the utilization of blood agar. Concurrently, the pH values of the culture media were measured at 0, 2, 4, 6, and 20 h.

### 2.5. Assessment of Morphology of C. albicans

*C. albicans* morphological changes were evaluated by observing the planktonic culture medium at 20 h using a light microscope (Olympus BX43, 214, Tokyo, Japan) with a 100X oil objective (Olympus UPlanFL N 100X, Tokyo, Japan). A volume of 20 µL of the culture medium was deposited on a glass slide and observed without staining.

### 2.6. Quantitative Real-Time Polymerase Chain Reaction (qRT-PCR)

At 20 h, 4 mL culture suspensions from the above planktonic model were collected for RNA exaction. Complementary DNA was synthesized by using 0.2 µg of purified RNA as a template and the BioRad iScript cDNA synthesis kit (Bio-Rad Laboratories, Inc., Hercules, CA, USA). Applied Biosystems^TM^ PowerTrack^TM^ SYBR Green Master Mix and a QuantStudio^TM^ 3 Real-Time PCR System were utilized to amplify cDNA and negative control samples (Thermo Fisher Scientific, Wilmington, DE, USA). In each 20 µL reaction, cDNA, 10 µM of each primer, and a 2× SYBR-Green mix (SYBR-Green and Taq DNA Polymerase) were present. *gyrA* [28], *ACT1*, and *rpob* were used as internal reference genes for *S. mutans*, *C. albicans*, and *L. plantarum,* respectively. The comparative CT method was applied to the data analysis [29]. The genes and primers used are listed in Table S1.

### 2.7. Statistical Analysis

Statistical analyses were performed using SPSS Statistics, version 28 (SPSS Inc., Chicago, IL, USA). CFU values were converted to natural log values, and zero values remained unchanged. Shapiro–Wilk normality tests with a 95% confidence interval were used to examine if data sets were normally distributed. For data exhibiting normal distribution, the comparisons between two groups were conducted using a *t*-test, while one-way ANOVA, followed by a post hoc test, was used for comparisons involving more than two groups. For data exhibiting non-normal distribution, Mann-Whitney was employed for two groups comparison, and Kruskal-Wallis was used for comparisons of more than two groups. A *p*-value less than 0.05 was considered statistically significant.

## 3. Results

### 3.1. Impact of Prebiotics GOS on Individual Growth of S. mutans, C. albicans, and L. plantarum

**Figure 1:**
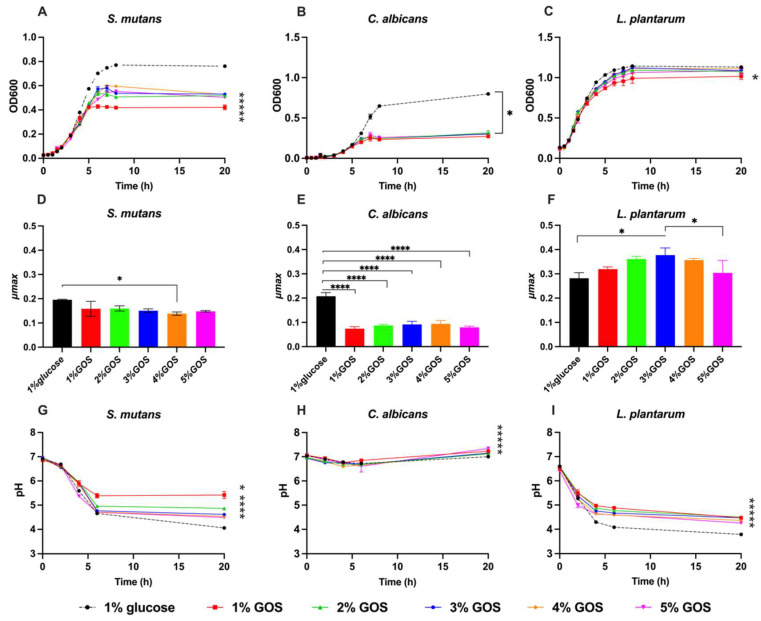
Single species growth with glucose or GOS sugar substrate. (**A**–**C**) Growth curves for single species of *Streptococcus mutans*, *Candida albicans*, *and Lactobacillus plantarum* 14,917 in TSBYE with 1–5% GOS or 1% glucose. (**D**–**F**) The maximal specific growth rate (µmax) for single species of *S. mutans*, *C. albicans*, *and L. plantarum* 14,917 in TSBYE with 1–5% GOS or 1% glucose. (**G**–**I**) pH changes during the above growth progress. * Indicate that the maximum plateau OD, µmax, or pH at 20 h with GOS, were significantly different from that with 1% glucose (*p* < 0.05). **** *p* < 0.0001.

By monitoring the optical density (OD) at 600 nm over the period of 20 h, the growth of *S. mutans* UA159, *C. albicans* 5314, and *L. plantarum* ATCC 14,917 strains in the presence of 1–5% GOS and 1% glucose was evaluated. Figure 1 displays the growth curves, µmax, and pH variations. 

GOS significantly impeded the growth of *C. albicans* and *S. mutans* in terms of growth amount and growth speed. However, GOS did not impact the growth of *L. plantarum* 14,917. GOS, as the sole carbohydrate source of *S. mutans*, *C. albicans*, *and L. plantarum* 14,917, yielded a more neutral culture medium pH.

Notably, Figure 1B shows the very limited growth of *C. albicans* in GOS compared to that in glucose condition. The ODs at the end of the exponential phase of *C. albicans* growth in GOS (at 7 h) was around 0.25, and they only increased to 0.3 at the end time point (at 20 h). While in glucose condition, the OD was much higher. It was over 0.7 at the end of the exponential phase (at 8 h) and thereafter. The µmax of *C. albicans* in GOS were significantly reduced compared to that in glucose condition (*p* < 0.0001 for all the concentration of GOS) (Figure 1E).

*S. mutans* could grow in GOS (Figure 1A), and the ODs reached 0.5 at end of the exponential phase (at 7 h) and thereafter. However, its growth in GOS could not reach OD 0.8 as in the glucose condition. The maximum plateau OD of GOS-grown *S. mutans* was substantially lower than that of glucose-grown *S. mutans*. Particularly in the 1% GOS condition, *S. mutans* exhibited the least quantity of growth compared to that grew in 1% glucose (*p* < 0.0001). In addition, the µmax of GOS-grown *S. mutans* were lower than that of glucose-grown ones.

In contrast, when grown with 1–5% GOS, *L. plantarum* 14,917 generally grew as well as it did in glucose condition with comparable maximum plateau OD (Figure 1C) (*p* > 0.05), except 1% GOS, and comparable µmax (Figure 1F). Interestingly, the µmax of 3% GOS-grown *L. plantarum* 14,917 (0.377 ± 0.030 h^−1^) was significantly higher than glucose grown (0.282 ± 0.023 h^−1^) (*p* = 0.014).

Figure 1G–I shows the pH changes during the single species growth. In general, the medium pH for *L. plantarum* 14,917 (Figure 1I), *S. mutans* (Figure 1G), and *C. albicans* (Figure 1H) were significantly higher in GOS conditions than that in the glucose condition. There are dose-dependent effects of the medium pH for *L. plantarum* 14,917 and *S. mutans* at various GOS concentrations. 1% GOS yielded the highest pH, with the pH value of 4.5 and 5.4 for *L. plantarum* 14,917 and *S. mutans*, respectively, which are significantly higher than those (pH 3.8 and 4.1, respectively) in the 1% glucose condition.

### 3.2. The Impact of GOS on S. mutans-C. albicans Duo-Species Growth

**Figure 2:**
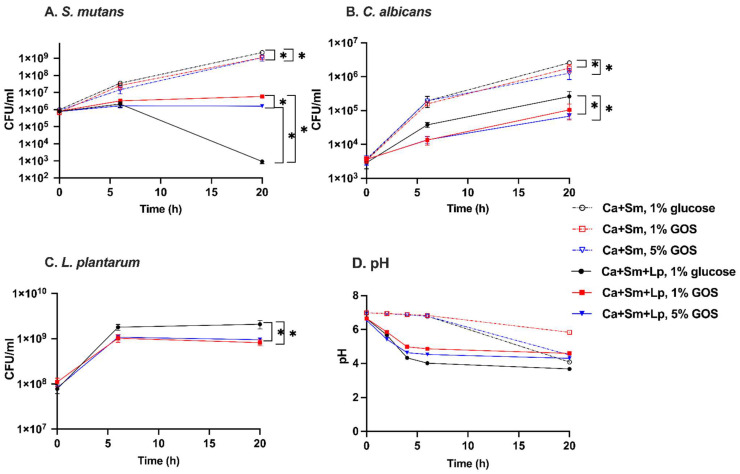
Interactions in duo-species (control) and *L. plantarum* treated groups in planktonic condition. (**A**–**C**) The growth of *S. mutans*, *C. albicans,* and *L. plantarum* 14,917 in planktonic condition is plotted. (**D**) pH changes of duo-species and *L. plantarum* 14,917 treated groups in TSBYE with 1, 5% GOS or 1% glucose. ‘Ca + Sm’ referred to the cocultivation of *C. albicans* and *S. mutans.* ‘Ca + Sm + Lp’ referred to *C. albicans* and *S. mutans* duo-species treated with *L. plantarum* 14,917. Viable cells were measured to determine the inhibitory effects. * Indicate that *p* < 0.05 when comparing the two groups.

The growth of *S. mutans* with 1% GOS and 5% GOS was inhibited by about 50% at 20 h compared to that with 1% glucose (*p* < 0.0001 for 1% GOS; *p* = 0.0015 for 5% GOS) (Figure 2A). Moreover, 1% and 5% GOS reduced the growth of *C. albicans* by about 30 and 50% at 20 h, respectively (*p* = 0.0048 for 1% GOS; *p* < 0.001 for 5% GOS). However, there is no statistically significant difference regarding the potency of the inhibitory effect on *S. mutans* or *C. albicans* between 1% and 5% GOS. For the first 6 h, the pH changes of the three different substrates’ culture media declined to a comparable degree. At 20 h, GOS as substrates created a higher medium pH than 1% glucose (*p* < 0.0001). The 1% GOS condition displayed the highest culture medium pH of 5.83 at 20 h.

### 3.3. The Impact of GOS on Inhibitory Capacity of L. plantarum against S. mutans-C. albicans Duo-Species Growth 

The growth of *S. mutans* and *C. albicans* was significantly repressed by *L. plantarum* 14,917 in utilization of 1% and 5% GOS at 6 and 20 h (Figure 2A,B) compared to duo-species with GOS. GOS enhanced the anti-fungal performance of *L. plantarum* 14,917 on *C. albicans* at 6 and 20 h (*p* < 0.0001 for both 1% and 5% GOS). However, this enhanced effect was not influenced by the concentration of GOS (*p* > 0.05). GOS did not augment the anti-bacterial effect of *L. plantarum* 14,917 compared to glucose. However, *L. plantarum* 14,917 with 5% GOS was more effective against *S. mutans* than that with 1% GOS at 20 h (*p* < 0.0001) (Figure 2A). The inhibitory effect on *S. mutans* under 5% GOS condition and 1% glucose at 6 h were similar (*p* > 0.05) and higher than 1% GOS (*p* < 0.05). 

In the *L. plantarum* 14,917 treated duo-species model, using GOS as the carbohydrate substrate led to a higher culture medium pH than 1% glucose. Particularly, 1% GOS led to the highest culture medium pH of 4.6 at 20 h (Figure 2D). 

### 3.4. GOS Facilitated L. plantarum’s Inhibition of C. albicans Hyphae Formation

**Figure 3:**
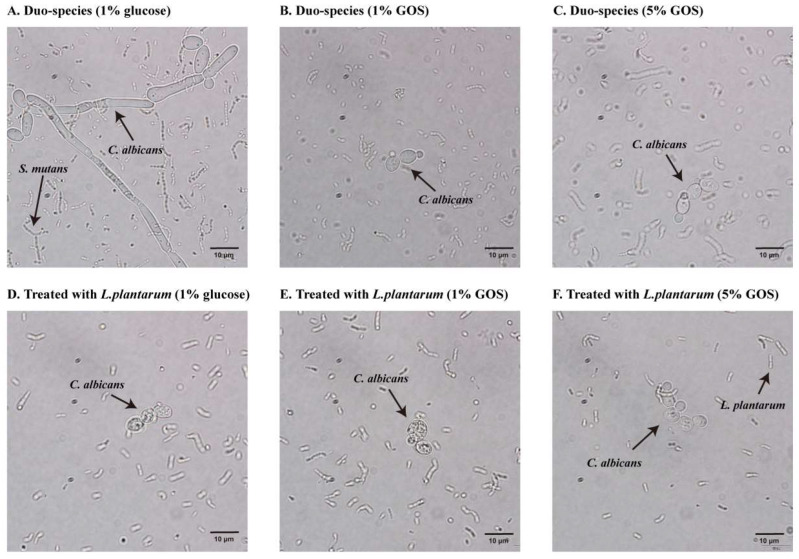
Form of *C. albicans* treated with GOS and *L. plantarum* 14,917. (**A**) *S. mutans* and *C. albicans* duo-species grown in 1% glucose at 20 h. (**B**) *S. mutans* and *C. albicans* duo-species grown in 1% GOS at 20 h. (**C**) *S. mutans* and *C. albicans* duo-species grown in 5% GOS. (**D**) *S. mutans* and *C. albicans* duo-species grown in 1% glucose with the addition of *L. plantarum* 14,917 at 20 h. (**E**) *S. mutans* and *C. albicans* duo-species grown in 1% GOS with the addition of *L. plantarum* 14,917 at 20 h. (**F**) *S. mutans* and *C. albicans* duo-species grown in 5% GOS with the addition of *L. plantarum* 14,917 at 20 h.

Figure 3 reveals that the transition from yeast to hyphal or pseudohyphal form of *C. albicans* was inhibited by GOS alone and the combination of *L. plantarum* 14,917 and GOS.

### 3.5. Expression of Genes of Interest in Mixed-Species Model

The impact of exposure to GOS and *L. plantarum* 14,917 on *C. albicans* and *S. mutans* was further assessed and compared using qRT-PCR. 

**Figure 4:**
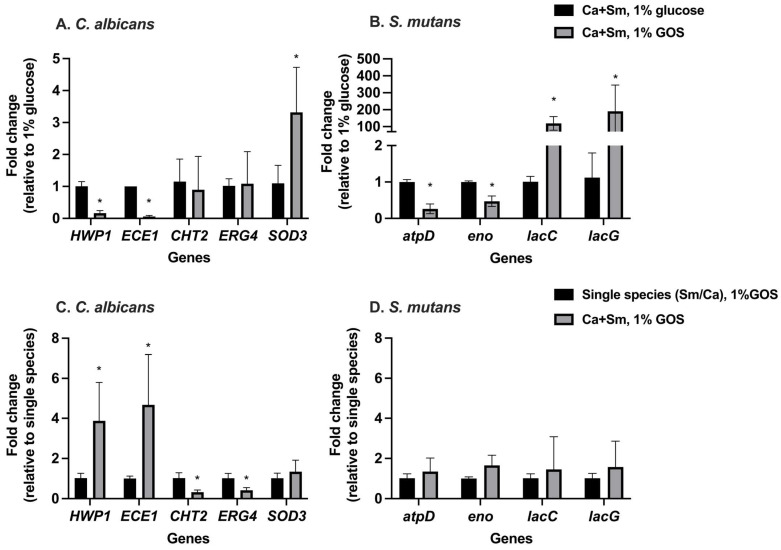
Expression of *C. albicans* and *S. mutans* genes in single and duo-species culture at 20 h. (**A**) *C. albicans* genes expression in duo-species with 1% GOS relative to that with 1% glucose. (**B**) *S. mutans* genes expression in duo-species with 1% GOS. (**C**) *C. albicans* genes expression in duo-species with 1% GOS relative to *C. albicans* grown solely with 1% GOS. (**D**) *S. mutans* genes expression in duo-species with 1% GOS relative to *S. mutans* grown solely with 1% GOS. * Indicate that the expression of genes in the duo-species with 1% GOS was significantly different from that in the duo-species with 1% glucose or single species with 1% GOS (*p* < 0.05).

Compared to 1% glucose, GOS alone reduced the expression of most *C. albicans virulence* genes in *S. mutans-C. albicans* duo-species model, shown in Figure 4A. *HWP1* and *ECE1*, associated with hyphal growth and adhesion to host cells, were 6.2-fold and 16.3-fold down-regulated, respectively (*p* < 0.0001). Additionally, *SOD3*, a gene involved in oxidative stress response, was significantly up-regulated (*p* = 0.0377). However, *CHT2*, the gene involved in chitinase encoding, was slightly down-regulated (*p* > 0.05). Whereas, the expression of *S. mutans genes*, *atpD* (stress response gene related to *ATPase* complex and acid tolerance) and *eno* (associated with degradation of carbohydrates via glycolysis), were significantly reduced by 2.8 fold (*p* < 0.0001) and 1.1 fold (*p* = 0.0005), respectively, in GOS condition, compared to duo-species in glucose. Moreover, *lacC and lacG*, the genes involved in galactose metabolism, were significantly up-regulated (*p* < 0.05) compared to *S. mutans* grown in glucose (Figure 4B).

Compared to 1% GOS-grown single species, gene expression of *C. albicans* and *S. mutans* in duo-species in 1% GOS was exhibited (Figure 4C,D). Notably, in 1% GOS condition, with the presence of *S. mutans*, *C. albicans* genes *HWP1* and *ECE1* were significantly up-regulated (*p* < 0.05). In addition, *CHT2* and *ERG4* genes were down-regulated (*p* < 0.01) (Figure 4C). All the virulence genes of *S. mutans* were up-regulated when co-cultured with *C. albicans*, compared to *S. mutans* grew individually, despite no statistically significant difference (Figure 4D).

**Figure 5:**
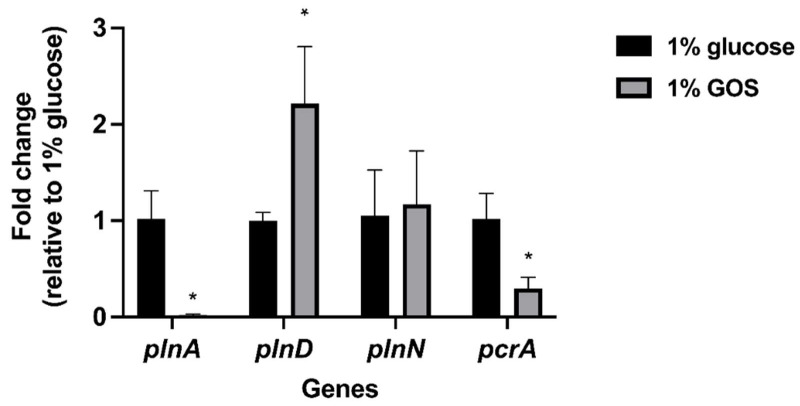
Expression of *L. plantarum* genes in single-species model at 20 h. * Indicate that the expression of genes of *L. plantarum* 14,917 with 1% GOS was significantly different from that of *L. plantarum* 14,917 with 1% glucose (*p* < 0.05).

The gene expression of *L. plantarum* 14,917 grown alone with 1% GOS is shown in Figure 5. *plnA*, encoding plantaricin, was down-regulated (*p* < 0.0001), compared to *L. plantarum* 14,917 grown alone with glucose. However, *plnD*, another gene encoding plantaricin, was significantly up-regulated (*p* = 0.0402). In addition, there was an up-regulation of *plnN* gene, but the difference was not statistically significant.

**Figure 6:**
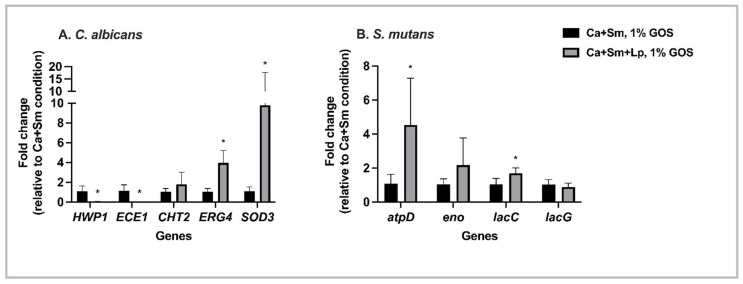
Genes expression in duo- and multi-species culture at 20 h. (**A**) *C. albicans* genes expression in duo-species treated with *L. plantarum* 14,917 in utilization of 1% GOS relative to duo-species with 1% GOS. (**B**) *S. mutans* genes expression in duo-species treated with *L. plantarum* 14,917 in utilization of 1% GOS relative to duo-species with 1% GOS. * Indicate that the expression of genes in the duo-species treated with *L. plantarum* 14,917 in 1% GOS was significantly different from duo-species in 1% GOS (*p* < 0.05).

We explored the gene expression in *S. mutans* and *C. albicans* duo-species treated with *L. plantarum* 14,917 in utilization of 1% GOS (Figure 6). In 1% GOS condition, compared to the *S. mutans* and *C. albicans* duo-species, the *L. plantarum* 14,917 treated group had significant down-regulations of *C. albicans* genes involved in hyphal growth (*HWP1* and *Ece1*): *HWP1* with 22.9-fold change (*p* = 0.0008), and *ECE1* with 55.8-fold change (*p* = 0.0012). *ERG4*, and *SOD3* genes were up-regulated (*p* < 0.05) (Figure 6A). The *lacG* gene of *S. mutans* was down-regulated in *L. plantarum*-GOS treated group compared to the none-treated duo-species model (Figure 6B). In addition, *atpD* and *lacC genes* were significantly up-regulated (*p* < 0.05).

## 4. Discussion

Given that few studies have evaluated the efficacy of *L. plantarum* 14,917 utilizing GOS on inhibiting *C. albicans* and *S. mutans*, our findings shed light on caries control from a novel ecological perspective that utilizes prebiotics and probiotics. The results of the present investigation indicate that GOS alone can contribute to a more neutral pH, that is higher than 5.5, even in a high-caries risk model, and utilizing GOS as the carbon source can enhance the anti-fungal activity of *L. plantarum* 14,917 against *C. albicans.*

### 4.1. Properties and Benefits of GOS 

GOS is characterized by a mix of structures that exhibit variations in the degree of polymerization and glycosidic linkage between the galactose moieties or between galactose and glucose. 

In addition to its prebiotic properties, GOS exhibited non-digestibility, good stability, a high capacity to hold moisture, high solubility, and a low glycemic index [30,31]. It has a sweetness of typically 0.3 to 0.6 times that of sucrose [30]. In addition, the safety of GOS has been acknowledged in various nations. In the United States, GOS is generally recognized as safe (GRAS), in the European Union as non-novel foods, and in Japan as foods for specific health use (FOSHU) [30]. Additionally, GOS was permitted in infant and follow-on formulas by the Food Standards of Australia and New Zealand (FSANZ) in 2008.

GOS can markedly promote the growth of *Bifidobacteria* and *Lactobacilli *in the colon and produce short-chain fatty acid (SCFA) from fermentation [31,32]. Hence, it is widely used as a low-calorie sweetener in fermented milk products, confections, bread, and beverages [30,31]. Extensive studies have been conducted regarding the systemic health advantages of GOS. Comparatively, few studies have examined the effectiveness of GOS on oral health.

### 4.2. GOS, a Potential Anti-Caries Agent 

In the present study, GOS as the sole carbohydrate source resulted in a less acidic environment for the single-, duo- and multispecies planktonic models of *C. albicans*, *S. mutans,* and *L. plantarum* 14,917. A noteworthy finding is that the pH was 5.8 in *C. albicans*-*S. mutans* duo-species models in the utilization of 1% GOS, which is above the well known critical pH for enamel dissolution of 5.5. Moreover, hyphal formation genes (*HWP1* and *ECE1*) of *C. albicans* and acid stress genes (*atpD*) of *S. mutans* in duo-species model, which mimicked high caries risk condition, with 1% GOS was significantly repressed compared to glucose. This finding indicates that GOS may act as a disincentive to hyphal formation and acid resistance of *C. albicans*-*S. mutans* duo-species medium. Hence, GOS has anti-caries potential, considering the ability to neutralize the environment pH and the ability to disturb the virulence genes expression of *C. albicans*-*S. mutans* duo-species medium.

Interestingly, single-species growth screening revealed that *C. albicans* and *S. mutans* could not grow as effectively in GOS as in 1% glucose. Of note is that, when we cocultured *C. albicans* and *S. mutans*, GOS alone did not appear to effectively inhibit the development of *C. albicans* and *S. mutans* in terms of CFU/mL. In addition, compared to single-species planktonic models, *S. mutans genes *involved in energy metabolism (*atpD*) and carbohydrate metabolism (*eno*, *lacC* and *lacG*) were up-regulated with the presence of *C. albicans.* The trend was similar with *S. mutans* and *C. albicans* in utilization of sucrose [33]. The metabolism of *S. mutans* may be fostered when grown together with *C. albicans*. Similarly, hyphal formation genes (*HWP1* and *ECE1*) and SOD3, related to tolerance to oxidative stress, were induced when grown with *S. mutans.* Those findings indicate that the symbiotic relationship and cross-feeding effects between *S. mutans* and *C. albicans* exist when GOS is the sole carbohydrate. 

*LacA* (encoding β-galactosidase), *lacS* (encoding permease LacS), and *lacR* (a divergently oriented regulator) genes were essential in the utilization of GOS [23,24] by *Lactobacilli*. We speculate that GOS metabolites produced by *C. albicans* can be efficiently utilized by *S. mutans*, which may lack the permease LacS to aid in transporting highly polymerized molecules, such as GOS. *S. mutans* may also feed back *C. albicans* through the breakdown of disaccharides into monosaccharides [34]. This may explain the significant up-regulation of *lacC* and *lacG* genes in duo-species with GOS compared to that with glucose. Further research is needed to better understand the synergistic relationship between the metabolites produced by *S. mutans* and *C. albicans* when metabolizing GOS, as no study has yet reported relevant information on this topic.

### 4.3. Synbiotics Effect of GOS and L. plantarum 14,917 on Caries Control

To determine whether utilizing GOS could impact the antibacterial and antifungal capacity of *L. plantarum* 14,917, we treated *S. mutans* and *C. albicans* duo-species with *L. plantarum* 14,917. Our finding exhibited that the combination of GOS and *L. plantarum* 14,917 significantly suppressed the growth of *C. albicans* by around 1 log compared to *L. plantarum* 14,917 with glucose at 20 h. *L. plantarum* 14,917 in utilization of GOS showed a significantly inhibitory ability on *S. mutans* growth in comparison to the control group, which co-cultured *C. albicans* and *S. mutans.* However, the performance of *L. plantarum* 14,917 with 1% glucose still showed the best inhibitory effect on *S. mutans.*

In the current study, a significant down-regulation of the *pln A* genes and a significant up-regulation of *plnD* gene, responsible for encoding antimicrobial peptide plantaricin were observed in *L. plantarum* 14,917 with GOS in comparison to glucose. As plantaricin production of *L. plantarum* is based on the complicated operons system [35,36,37], the impact of GOS on plantaricin production remains unclear. Future research aimed at measuring plantaricin production could aid in clarifying the GOS effects of *L. plantarum*.

Relative to 1% glucose, the culture medium with GOS maintained a more neutral pH by roughly one pH unit. This significant pH difference might explain the dissimilar potency of the inhibitory effect of *L. plantarum* 14,917 under GOS and glucose. *S. mutans* is acidogenic and aciduric. These capacities help *S. mutans* survive under acidic conditions and give it a significant competitive edge against other bacterial species inhabiting dental plaque in acidic conditions [38,39,40]. However, extreme pH could pose a risk to acid-sensitive molecules, such as DNA and the metabolic machinery. Previous studies indicated *S. mutans* could continue to grow in continuous cultures at pH values, ranging from 4.5 to 5.0 [39,41]. Conversely, a pH below 4 could impact the viability of *S. mutans.* The cell membranes of *S. mutans* GS-5 were damaged by acidification, and *S. mutans* were killed at a pH lower than 4 [40]. Boisen et al. observed that the viability of *S. mutans* UA159 at pH 3.5 was significantly decreased to roughly 5% in planktonic culture [41]. However, the pH threshold for a significant impact of viability on *S. mutans* was not conclusive. Glucose was found to be protective against acid killing of *S. mutans* [42]. For this current study, carbohydrate exhaustion at 20 h, combined with low pH value, might contribute to the dramatic decrease in viable cells of *S. mutans* in glucose. 

With respect to *L. plantarum*, pH contributes to *L. plantarum*’s antimicrobial effect against *S. mutans*. Wasfin et al. revealed neutralization of culture supernatant of *L. plantarum* 14,917 to pH 6.5 significantly reduced the inhibitory effect on *S. mutans* isolated from caries dentin. This study indicated that acid contributes with other antimicrobial agents, such as hydrogen peroxide, bacteriocin, and biosurfactant, to inhibit growth [43]. 

## 5. Conclusions

GOS significantly inhibited the growth of *C. albicans* and *S. mutans* in terms of growth quantity and speed. Moreover, GOS enhanced the anti-fungal ability of *L. plantarum* 14,917 against *C. albicans* and helped to build a more neutral pH environment in high-risk carries’ planktonic conditions. Future studies are required to analyze the metabolism of GOS by *S. mutans* and *C. albicans* and the GOS effect using biofilm models.

## Data Availability

All data generated or analyzed during this study are included in this article. Further enquiries can be directed to the corresponding author.

## Supplementary Materials

The following supporting information can be downloaded at: https://www.mdpi.com/article/10.3390/nu15092017/s1, Table S1. Primers used in RT-qPCR.
