# Supplementary material for: Anti-cariogenic Properties of Lactobacillus plantarum in the Utilization of Galacto-Oligosaccharide
Source: Nutrients. 2023 Apr 22;15(9):2017. doi: 10.3390/nu15092017 (PMC10180963; doi:10.3390/nu15092017)
Supplement: Supplementary file 1 [file nutrients-15-02017-s001.zip › Table S1.pdf]

Table S1. Primers used in RT-qPCR [14].

Genes	Primers	Sequence	Amplicon Size
atpD	Sm_atpD_F	TGTTGATGGTCTGGGTGAAA	176
	Sm_atpD_R	TTTGACGGTCTCCGATAACC	
eno	Sm_eno_F	CAGCGTCTTCAGTTCCATCA	194
	Sm_eno_R	TCACTCAGATGCTCCAATCG	
lacG	Sm_lacG_F	ATTGGATGCGTGCTTTTGATGG	94
	Sm_lacG_R	CGACCGACACCCTTAATCTGG	
lacC	Sm_lacC_F	GCTGGAATTACATCGGCTCTTGC	157
	Sm_lacC_R	CCTCCGCTACCTCAATTTGTTGG	
ACT1	Ca_ACT1_F	TGCTCCAGAAGAACACCCA	182
	Ca_ACT1_R	CACCTGAATCCAAAACAATACCAGT	
HWP1	Ca_HWP1_F	TGGTGCTATTACTATTCCGG	182
	Ca_HWP1_R	CAATAATAGCAGCACCGAAG	
ECE1	Ca_ECE1_F	GCTGGTATCATTGCTGATAT	168
	Ca_ECE1_R	TTCGATGGATTGTTGAACAC	
CHT2	Ca_CHT2_F	TTGGGATGCTTCTGGGGCTT	111
	Ca_CHT2_R	GCAGAAGAAGATGGGGCAACAC	
ERG4	Ca_ERG4_F	TCAAATGTGCCAATGGTTCT	101
	Ca_ERG4_R	AGCCCAAGTCAATGTTTGAA	
SOD3	Ca_SOD3_F	CAGTATGGGTCTGTTTCAAACCTTA	211
	Ca_SOD3_R	GATATTGCAAGTAGTACGCATGTTC	
rpoB	Lp14_rpoB_F	CACCGTACCCGTAGAAGTTATGC	106
	Lp14_rpoB_R	GGAGACCTTGATCCAAGAACCA	
plnA	Lp14_plnA_F	GTGGAAAGAGTAGTGCGTATTC	135
	Lp14_plnA_R	CGCCATCTATACGAAATATAACTTG	
plnD	Lp14_plnD_F	TGAGGACAAACAGACTGGAC	415
	Lp14_plnD_R	GCATCGGAAAAATTGCGGATAC	
plnN	Lp14_plnN_F	ATTGCCGGGTTAGGTATCG	146
	Lp14_plnN_R	CCTAAACCATGCCATGCAC	
